# Plant-Expressed Receptor Binding Domain of the SARS-CoV-2 Spike Protein Elicits Humoral Immunity in Mice

**DOI:** 10.3390/vaccines9090978

**Published:** 2021-09-01

**Authors:** Puna Maya Maharjan, Jinyeong Cheon, Jiyun Jung, Haerim Kim, Jaewon Lee, Minjeong Song, Gi Uk Jeong, Youngchan Kwon, Byoungshik Shim, Sunghwa Choe

**Affiliations:** 1 G+FLAS Life Sciences, 123 Uiryodanji-gil, Osong-eup, Heungdeok-gu, Cheongju-si 28161, Korea; punamaya.maharjan@gflas.com (P.M.M.); 5271248@naver.com (J.L.); minjeong.song@gflas.com (M.S.); 2 School of Biological Sciences, College of Natural Sciences, Seoul National University, Gwanak-gu, Seoul 08826, Korea; 3 G+FLAS Life Sciences, 38 Nakseongdae-ro, Gwanak-gu, Seoul 08790, Korea; jinyeong.cheon@gflas.com (J.C.); jiyun.jung@gflas.com (J.J.); haerim.kim@gflas.com (H.K.); 4 Center for Convergent Research for Emerging Virus Infection, Korea Research Institute of Chemical Technology, Daejeon 34114, Korea; jay7@krict.re.kr (G.U.J.); yckwon@krict.re.kr (Y.K.); 5 International Vaccine Institute, SNU Research Park, 1 Gwanak-ro, Gwanak-gu, Seoul 08826, Korea; ByoungShik.Shim@ivi.int

**Keywords:** receptor binding domain (RBD), SARS-CoV-2, *Nicotiana benthamiana*, plant-based vaccine, humoral immunity, glycoengineering, neutralizing antibody, COVID-19, transient expression, plant specific glycosylation

## Abstract

The current 15-month coronavirus disease-19 (COVID-19) pandemic caused by SARS-CoV-2 has accounted for 3.77 million deaths and enormous worldwide social and economic losses. A high volume of vaccine production is urgently required to eliminate COVID-19. Inexpensive and robust production platforms will improve the distribution of vaccines to resource-limited countries. Plant species offer such platforms, particularly through the production of recombinant proteins to serve as immunogens. To achieve this goal, here we expressed the receptor binding domain (RBD) of the SARS-CoV-2 spike (S) protein in the glycoengineered-tobacco plant *Nicotiana benthamiana* to provide a candidate subunit vaccine. This recombinant RBD elicited humoral immunity in mice via induction of highly neutralizing antibodies. These findings provide a strong foundation to further advance the development of plant-expressed RBD antigens for use as an effective, safe, and inexpensive SARS-CoV-2 vaccine. Moreover, our study further highlights the utility of plant species for vaccine development.

## 1. Introduction

The recurrent outbreaks of coronavirus (CoV) infections over the last two decades caused by severe acute respiratory syndrome (SARS)-coronavirus-2 (SARS-CoV-2); SARS-CoV, 2002, China; and Middle East respiratory syndrome CoV (MERS-CoV), 2012, Saudi Arabia, have inflicted devastating health and socioeconomic impacts worldwide. Subsequent to its first identification in Wuhan, China in December 2019, the highly contagious SARS-CoV-2 spread rapidly throughout the world, causing at least 3.77 million deaths among 174.5 million confirmed cases as of 12 June 2021 [1]. The sequence of the 30-kb positive-sense single-stranded RNA genome of the novel SARS-CoV-2 is 96%, 80%, and 50% identical to those of the Bat coronavirus (Bat-CoV), SARS-CoV, and MERS-CoV, respectively [2,3,4]. The SARS-CoV-2 genome comprises 13–15 putative open reading frames that generate 11 proteins, including the structural transmembrane spike (S), envelope (E), membrane (M), and nucleocapsid (N) proteins, as well as six accessory proteins [5,6]. Despite high sequence identities among the genomes of these betacoronaviruses, their infection and fatality rates are highly variable, being approximately 2.16%, SARS-CoV-2; 15%, SARS-CoV; and 35%, MERS [1,7,8].

The membrane glycoprotein called the spike (S) protein, comprises the receptor binding protein S1 and the membrane fusion protein S2. Entry of the virus into its host cell requires the S protein, which involves attachment of S1 to the host cell followed by host–virus membrane fusion triggered by a conformational change of S2 [9]. In the mature virus, the S protein forms a trimer with a head domain composed of three S1 subunits atop a trimeric S2 stalk [10]. Virus entry into cells initiates upon binding of the S1-receptor binding domain (RBD) to angiotensin converting enzyme 2 (ACE2) on the cell surface. Furthermore, the RBD binds to ACE2 only when present in a standing position [9,11,12]. The SARS-CoV-2 RBD binds ACE2 with 10 times higher affinity than the SARS-CoV RBD, contributing to efficient cell entry of the former [13].

The rapid and uncontrollable transmission of COVID-19 among a large population urgently requires highly effective vaccines, other therapeutics, and management strategies. Commercial vaccines are typically produced using egg-based systems or microbial and mammalian cells. However, these production systems have shortcomings such as long production times, animal-associated safety issues, requirement for complex facilities, and high cost [14]. Alternatively, plant species serve as a promising platform that assures low production cost, safety, rapid production, and production scalability [14,15,16]. Moreover, vaccines can be produced using abundant and edible plants such as the potato and tomato [17,18].

The tobacco plant (*Nicotiana tabacum*) was the first plant used to produce monoclonal antibodies [19]. Subsequently, the quantity and quality of plant-based biopharmaceuticals have greatly improved [20,21]. Advances in the plant production platform include the development of high-yield protein expression vector systems, expansion of the diversity of host plants, and glycoengineering of host plants to enhance drug efficacy [22,23,24,25]. Present plant platforms produce diverse pharmaceuticals such as recombinant antibodies, enzymes, vaccines, hormones, growth factors, and other therapeutics, and clinical trials and commercial production are ongoing [24,26,27,28,29,30,31].

The first plant-made pharmaceutical approved for human use by the United States Food and Drug Administration was carrot cell-produced taliglucerase (Elelyso) for treating Gaucher disease. This enzyme-replacement therapeutic is more effective than the equivalent molecule produced using the Chinese hamster ovary cell line [21,32,33]. The tobacco plant-based monoclonal antibody cocktail ZMapp was administered to people infected with Ebola virus during the 2014 outbreak and is currently the subject of a phase II clinical trial [34,35]. Recently, a phase III clinical trial of a quadrivalent vaccine for season influenza produced using the tobacco plant was completed [36]. Moreover, plant-based pandemic influenza vaccine, rotavirus vaccine are at phase II and phase I clinical trial, respectively [37]. The potential applications of plant production platforms to fight COVID-19 are the subjects of numerous publications [30,38,39,40,41,42,43,44,45]. Plant-based COVID-19 vaccines, CoVLP developed by Medicago and KBP201 developed by Kentucky bioprocessing have entered clinical trial phase III and phase I/II, respectively [46,47,48].

The RBD, as well as the S protein, are major targets for the development of vaccines and other antiviral therapeutics because of their high immunogenicities and their requirement for virus entry into cells. Furthermore, the glycosylation pattern of an antigen influences its surface binding affinity, antigenicity, and efficacy as a vaccine [49,50,51]. Antigens with different glycosylation patterns and antigenicities are produced in plants using molecularly engineered glycosylation pathways [52]. Here we report the expression of the RBD antigen in plants, which was glycoengineered through inactivation of plant-specific alpha-(1,3)-fucosyltransferase and beta-(1,2)-xylosyltransferase. This plant-based RBD antigen efficiently induced the production of RBD-specific IgG antibodies in mice that neutralized the infectivity of SARS-CoV-2 in Vero E6 cell. Such a subunit vaccine can be inexpensively produced to address the economic burden of mass immunization of the populations of resource-limited countries.

## 2. Materials and Methods

### 2.1. Plasmid Construction

**Figure 1:**
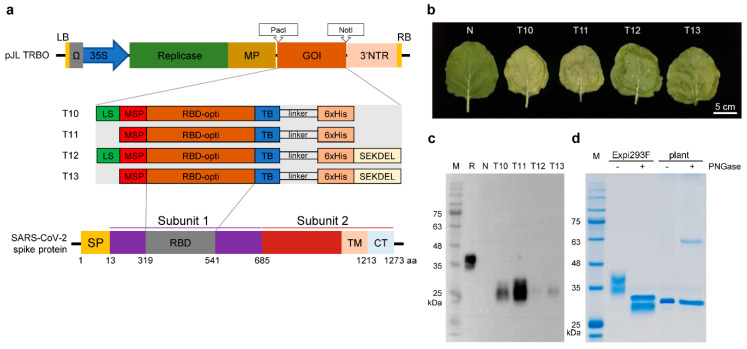
Plant-based SARS-CoV-2 RBD from *Nicotiana benthamiana* with engineered glycan pattern: (**a**) Schematic representation of T-DNA region of four different constructs harboring SARS-CoV-2 RBD. The constructs were made with addition of extra sequences at 5′ (MSP or LS and MSP) and 3′ (6xHis or 6xHis and SEKDEL) of RBD. Abbreviations: LB, left border T-DNA; RB, right border T-DNA; 35S, CaMV 35S promoter; Ω, TMV leader sequence; Replicase: TMV replicase; MP, TMV movement protein; GOI, Gene of interest; 3′NTR, TMV 3′ nontranslated region; LS, leader sequences of SARS-CoV-2 spike protein; MSP, Murine signal peptide; RBD-opti, RBD plant codon optimized; TB, thrombin protease cleavage site; linker, SSGSSG space linker; 6xHis, hexa histidine tag; SEKDEL, ER retention signal peptide sequences. (**b**) Sample 4 DPI leaves expressed four kinds of RBD constructs in glycoengineered *Nicotiana benthamiana*. (**c**) Immunoblot analysis of total soluble proteins extracted from 4 DPI leaves. (**d**) SDS-PAGE of deglycosylated RBDs. Deglycosylation of Expi293F-based RBD by PNGase F causes a band shift from ca. 35 kDa to ca. 30 kDa. Plant-based RBD stayed 30 kDa regardless of whether the peptide-N-glycosidase (PNGase) was treated or not. The bands at ca. 36 kDa and ca.64 kDa correspond to the enzyme PNGase F and PNGase A, respectively. Abbreviations: M, protein size marker; R, 100 ng of SARS-CoV-2 RBD protein from Expi293F cells (positive control); N, non-infiltrated (negative control); DPI, days post infiltration.

The nucleotide and amino acid sequences of the SARS-CoV-2 spike protein (S) were obtained from the United States National Center for Biotechnology Information. The nucleotide sequences encoding amino acid residues 319–541 of the S protein (Protein ID YP_009724390.1) corresponding to its RBD were codon-optimized to improve expression in *N. benthamiana* and synthesized by GenScript, Piscataway, NJ, USA. The DNA sequences encoding the RBD were PCR-amplified using specific primer sets to generate the various constructs as presented in Figure 1. Amplicons were cloned into a plant expression vector, pJL-TRBO using the designated cloning site with NEBuilder HiFi DNA Assembly Master Mix (New England Biolabs, Iswich, MA, USA). Correct insertion of a construct into the plasmid was confirmed using colony PCR and Sanger sequencing. pJL-TRBO was received from Addgene repository where it was deposited by John Lindbo, https://www.addgene.org/80082/ (accessed on 13 June 2021) [53].

### 2.2. Agroinfiltration of N. benthamiana

We used a heat-shock method to transform *Agrobacterium tumefaciens* strain EHA105 with the RBD constructs (Figure 1). Agrobacterium cells were cultured in YEP medium containing kanamycin (100 mg/L) and rifampicin (25 mg/L) at 28 °C for 2 days. The cell suspension was diluted with infiltration buffer (10 mM 2-N-morpholinoethane sulfonic acid pH 5.6, 10 mM magnesium sulfate) to OD_600_ = 0.2. The glycoengineered *N. benthamiana* was grown in a hydroponic system for 4–5 weeks at 22 °C during the day, 24 °C at night, with a 16-h light/8-h dark cycle, in a closed, humidified sterile container (approximately 50% humidity). Agroinfiltration was performed using a syringe to inject individual leaves. For large-scale protein production and purification, agroinfiltration was performed by submerging the whole plant into infiltration medium. Infiltrated tobacco plants were grown for the 4–7 days before harvest in the recovery room.

### 2.3. Protein Expression Analysis

To measure target-protein expression, tobacco leaves were harvested 4–5 days after *Agrobacterium* infiltration and subjected to Western blot analysis as follows: Freshly harvested leaves (100 mg) were extracted with 50 mM Tris buffer, pH 8.8. The supernatant was transferred to a new tube, and the protein concentration was determined using the Bradford assay (BioRad). Soluble protein samples (20 μg) were loaded onto the lanes of a 10% Tris-glycine SDS-PAGE gel, which was stained using EasyStain (DG-GS1000, Dogenbio, Seoul, Korea) or electrophoretically transferred to a PVDF membrane. The membrane was probed with a rabbit polyclonal anti-SARS-CoV-2 (COVID-19) Spike RBD antibody (10–529, ProSci), and recombinant RBD was detected using ECL detection reagent. To determine protein yields, 100 ng RBD protein expressed in human Expi293F-cell using Expi293™ Expression System Kit (ThermoFisher Scientific, Waltham, MA, USA) was used as a positive control, and the amount of RBD was calculated by analyzing the percentage volume of band density vs. the positive control. Signal intensities were measured using a gel imaging system (UVITECH, Cambridge, UK).

### 2.4. Purification of Plant-Based RBD

Tobacco leaves were harvested 4-days post infiltration and mixed with precooled extraction buffer (10 mM sodium phosphate, pH 7.4), which was diffused into cells using vacuum infiltration, followed by 15 min incubation at 4 °C. The total soluble extract was prepared using a juicer. The crude soluble extract was clarified by centrifugation at 10,000 rpm for 10 min, followed by filtration through 0.45 μm and 0.20-μm filters (EATON, ALP-AG-30-0.45-3E and PARKER, ESBS-302-CAS, respectively). The filtered extract was loaded onto a 5-mL prepack His affinity column (HisTrap FF resin, Cytiva, 17-5255-01) and equilibrated with 10 column volumes (CV) of His buffer A (10 mM sodium phosphate, 5 mM imidazole, pH 7.4). The column was first washed with 15 CV of His buffer A and then with 15 CV His buffer A and 3% of His buffer B (10 mM sodium phosphate, 500 mM imidazole, pH 7.4). The bound protein was eluted with 30 CV of His buffers A and B using a linear gradient (3–50% His buffer B) with an ÄKTA Avant Chromatography system; and fractions were collected in absorbance ranged from 20 mAU to 50 mAU. The eluted fraction was added to a 5-mL prepacked SP column (SP sepharose column, Cytiva, 17-5054-01), equilibrated with 30 CV of SP buffer A (10 mM sodium phosphate, pH 7.4), washed with 15 CV SP buffer A, followed by elution using a linear gradient of 15 CV of SP buffers A and B (0–50%). The eluted fractions were pooled, and buffer exchange and determination of protein concentrations were performed using 1× PBS buffer in a 10-K molecular weight cutoff centrifuge tube (Amicon Ultra-4, Merck Millipore). The purity of purified recombinant RBD was evaluated using the Western blot method described above.

The concentration of purified RBD was determined using the BCA assay. SDS-PAGE was performed using a 10% Tris-glycine gel, which was stained using EZ-Gel Staining Solution (DG-GS1000, DoGenBio, Seoul, Korea) or electrophoretically transferred to a PVDF membrane. The membrane was probed using a mouse monoclonal SARS-CoV-2 spike RBD antibody (MAB10540, R&D systems) and an anti-6×His-HRP antibody (ab1187, Abcam). Recombinant RBD bound to the membrane was detected using ECL reagent and imaged using a chemiluminescence imaging system (MINI HD9, UVITEC, Cambridge, UK).

### 2.5. Protein Deglycosylation 

Deglycosylation of Expi293F-based and plant-based RBDs (2 µg) was performed using 1 µL of PNGase F or PNGase A (New England Biolabs, Ipswich, MA, USA), respectively. After deglycosylation, the samples were subjected to SDS-PAGE, and the gels were stained using EZ-Gel Staining Solution (DG-GS1000, DoGenBio, Korea) according to the manufacturer’s instruction.

### 2.6. LTQ-Orbitrap Mass Spectrometry

The purified plant-made RBD was separated in SDS-PAGE. The proteins in the gel slice were digested with trypsin at 37 °C overnight. After elution, the protein sample was separated on a C18 column (150 mm × 0.1 mm, pore size 3 μm, Agilent) with a linear gradient (A: 100% H_2_O, 0.1% formic acid and B: 100% ACN) at a flow rate of 300 mL/min. The mass spectra were analyzed using a dual-mass spectrometer (LTQ Orbitrap Velos, Thermo Scientific) coupled to a nano-LC system (EASY nLC, Thermo Scientific, Waltham, MA, USA). The mass data were obtained using a cycle combining one full MS scan (mass range 150–2000 *m/z*), and obtained proteins were identified by searching the MS/MS spectra using the tandem mass spectrometry database search program SEQUEST.

### 2.7. Mice and Immunization Protocol

Female BALB/cAnHsd mice of 18–20 g (5-weeks-old) were purchased from KOATECH (Korea), housed in an animal facility at the International Vaccine Institute (IVI, Korea), and acclimatized for 7 days prior to immunization. Studies were conducted in compliance with the guidelines of the IVI-Institutional Animal Care and Use Committee, which approved this study (IACUC, PN 2020-002). Mice were intramuscularly injected three times every 2 weeks with 10 μg of plant-based SARS-CoV-2 RBD after mixing with 25 μL of Imject Alum^TM^ adjuvant (77161, Thermo scientific), Alhydrogel^®^ adjuvant (vac-alu-250, Invivogen), or AddaVax^TM^ (vac-adx-10, Invivogen) to make total volume 50 μL. Control mice were injected with Imject Alum, Alhydrogel, or AddaVax. 5 mice were used in each test and control group. Blood samples were collected three times from the retro-orbital plexus 13 days after each injection, and sera were centrifugally separated from the blood and stored at −80 °C.

### 2.8. Enzyme-Linked Immunosorbent Assay (ELISA)

Expi293F-made RBD in PBS (50 μL per well of 1 μg/mL) was used to coat the wells of 96-well plates. After overnight incubation at 4 °C, the plates were washed three times with 200 μL of TBS buffer containing 0.05% Tween and then blocked with 100 μL of TBS containing 0.05% Tween and 5% skim milk per well at room temperature (RT) for 1 h. After washing once, 100 µL of sera diluted to 1:100 with TBS containing 0.05% Tween and 5% skim milk were added to each well. To measure titers, serially diluted sera (1/100 to 1/218,700) were added to the wells, and plates were incubated for 2 h at RT. After washing 5 times, HRP-conjugated anti-mouse IgG (1030-05, Southern Biotech, Birmingham, AL, USA), IgG1 (1070-05, Southern Biotech, Birmingham, AL, USA) or IgG2a (1080-05, Southern Biotech, Birmingham, AL, USA) diluted to 1:3000 with TBS containing 0.05% Tween and 5% skim milk was added, and plates were incubated at RT for 2 h. After washing five times, 100 μL of TMB/E (ES001, Millipore, Darmstadt, Germany) was added to each well. Reactions were stopped using 100 μL of 0.5 M HCl, and optical density was measured at 450 nm using a plate reader (FLUOstar Omega, BMG Labtech, Ortenberg, Germany). Expi293F-made RBD used in this experiment was expressed using Expi293™ Expression System (A14635, Thermo Fisher Scientific, Waltham, MA, USA).

### 2.9. Focus Reduction Neutralization Test (FRNT)

Vero (African green monkey kidney cell line; ATCC CCL-81) cells and Vero E6 (ATCC CCL-1586) cells were grown in high-glucose Dulbecco’s modified Eagle’s medium (DMEM; HyClone, Logan, UT, USA) in the presence of 10% fetal bovine serum (FBS; HyClone, Logan, UT, USA) and 100 U/mL of penicillin-streptomycin (Gibco, Carlsbad, CA, USA). SARS-CoV-2 (BetaCoV/Korea/KCDC03/2020) isolated from a patient in South Korea was obtained from Korea Centers for Disease Control and Prevention (KCDC) and propagated in Vero cells [54]. 

The neutralizing activities of mouse serum samples were determined using the FRNT_50_ assay [55]. Briefly, heat-inactivated mouse sera were serially diluted in Eagle’s minimum essential medium containing 2% FBS and incubated with 8 × 10^3^ plaque forming units for 1 h at 37 °C in a final volume of 50 μL. This mixture was added to 4 × 10^4^ Vero E6 cells (African green monkey kidney cells) in each well. After incubation for 8 h at 37 °C, the cells were washed, fixed, and then stained with the SARS-CoV-2 N-specific antibody (40143-R001, Sino Biological, Beijing, China) and a secondary horseradish peroxidase-conjugated goat antirabbit IgG (Biorad, Hercules, CA, USA). The signal was developed using an insoluble TMB substrate (Promega, Madison, WI, USA), and the number of infected cells was counted using an ImmunoSpot reader (CTL, Shaker Height, OH, USA). The serum dilution factor that achieved 50% neutralization (FRNT_50_) was calculated by nonlinear regression analysis GraphPad Prism 8.0; GraphPad Software, Inc., San Diego, CA, USA). All experiment involving infectious SARS-CoV-2 were performed in the biosafety level 3 (BSL-3) containment laboratory at Korea Research Institute of Chemical Technology (KRICT, Daejeon, Korea).

### 2.10. Statistical Analysis

All data were analyzed with GraphPad Prism 8.0 software. Statistically significant differences between two groups were determined using unpaired, two-tailed Student’s *t*-tests. *p* < 0.05 was considered to be statistically significant.

## 3. Results

### 3.1. Transient Expression of RBD in N. benthamiana 

To transiently express the RBD in *N. benthamiana*, we cloned the plant codon-optimized SARS-CoV-2 RBD sequences (amino acid residues 319–541) with additional sequences at the N- and C-termini into a tobacco mosaic virus (TMV)-based virus vector to generate the distinct RBD expression cassettes T10, T11, T12, and T13 (Figure 1a). 

To achieve high yields of RBD, an *Agrobacterium* harboring each RBD expression cassette was infiltrated into the leaves of the glycoengineered-FX *N. benthamiana* in which the genes encoding plant-specific alpha-(1,3)-fucosyltransferase (F) and beta-(1,2)-xylosyltransferase (X) were knocked out using CRISPR/Cas9 -based genome editing technology. The infiltrated tobacco leaves (4 days postinfiltration (DPI)) developed necrotic phenotypes, which were more severe for T10 and T11 compared with those of T12 and T13 (Figure 1b). The infiltrated leaves were harvested on DPI 4 to 7, and expression of the recombinant RBD was analyzed using standard SDS-PAGE and Western blot procedures (data not shown).

Protein bands migrated between 25 kDa and 35 kDa, which correspond to the predicted 27 kDa RBD band. Plant-based recombinant RBD was expressed from 4 DPI onward by all constructs, and constructs T10 and T11 expressed the highest levels (Figure 1c and Figure S1). Furthermore, the level of recombinant RBD was highest in the plant infiltrated with T11 on 4 DPI and gradually decreased (data not shown), and the concentration of T11–RBD in total soluble protein of the plant extract was 92 mg/kg fresh leaf weight on 4 DPI. Accordingly, we used T11 to produce the RBD.

Plant- and Expi293F-based RBDs migrated faster and slower, respectively, compared with the position of the 35 kDa marker. This may be explained by the lack of plant-specific fucose and xylose residues linked to the plant-based RBD or by differences between N- and O-glycosylation in human Expi293F cells vs. plant cells (Figure 1d). Treatment of Expi293F-based RBD with peptide-N-glycosidase (PNGase) F generated a band that migrated similarly to plant-based RBD, whereas plant-based RBD migrated similarly regardless of PNGase A treatment. These findings are likely attributable to the absence of N-glycan fucose and xylose in the -FX tobacco plant (Figure 1d). Furthermore, the higher relative molecular mass of plant-based RBD compared with 27 kDa predicted from its amino acid sequence is likely to be explained by its O-glycosylation [51].

### 3.2. Purification of Plant-Expressed RBD

**Figure 2:**
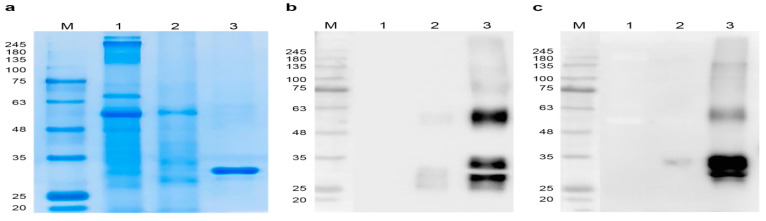
Purification of plant-based SARS-CoV-2 RBD Coomassie Blue staining (**a**), and Western blot of the SARS-CoV-2 RBD (**b**) and anti-6xHis (**c**) antibodies. M, protein size marker; Lane 1, non-infiltrated total soluble protein (TSP); Lane 2, RBD-infiltrated TSP; Lane 3, recombinant RBD purified from plant leaves.

The RBD, which was affinity-purified in two steps, that migrated through SDS-PAGE as a single (stained gels, Figure 2a) or 2 bands (in Western blots, Figure 2b,c) at 25–35 kDa, was detected using antibodies against the SARS-CoV-2 spike RBD and the His epitope tag (Figure 2b,c and Figure S2a,b). Moreover, N-terminal peptide sequencing using LC/LTQ-OrbiTrap MS confirmed the authenticity of the amino acid sequence of RBD excised from SDS-PAGE gels. The yield of purified RBD was 6 mg/kg fresh weight of starting material. The anti-RBD and epitope-tagged antibodies each detected a band corresponding to 64 kDa, consistent with an RBD dimer [56] (Figure 2b,c).

### 3.3. Plant-Based RBD Induces a Humoral Immune Response in Mice and Protects Vero E6 Cells from SARS-CoV-2 Infection

**Figure 3:**
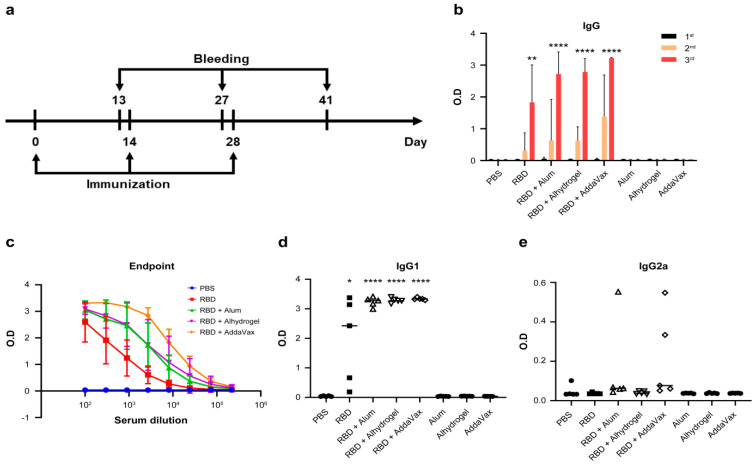
Serum antibody activities of mice immunized with the SARS-CoV-2 RBD: (**a**) Vaccination and blood collection scheme. (**b**) ELISAs of total IgG for first, second, and third collections of sera from the immunized mice. (**c**) RBD-specific IgG levels were determined using an endpoint dilution ELISA. (**d**,**e**) Isotypes, IgG1 (**d**) and IgG2a (**e**); ELISA of sera collected after the third immunization. Five mice were used in each test and control group. Serum antibody binding affinity was measured according to optical density at 450 nm determined using a microplate reader. Error bars show the mean ± SD. Statistically significant differences between two groups were determined using unpaired Student’s t-tests. *p*-values are shown for the sera collected from mice immunized three times with the RBD or RBD + adjuvant compared with mice injected with only PBS. * *p* < 0.05, ** *p* < 0.01,**** *p* < 0.0001.

To assess the immunogenicity of plant-based RBD in mice, we first compared the adjuvant effects of Alum, Alhydrogel, and AddaVax on the immune responses of mice to plant-based RBD (Table 1 and Figure 3a) [57]. ELISAs revealed that RBD-specific IgGs were present in the sera of RBD-immunized mice and that total IgG levels significantly increased after the third immunization (Figure 3b). In contrast, the ELISA titers of sera collected after the first dose of RBD and those of mice treated with PBS or adjuvant alone were negligible. Although plant-based RBD alone induced high ELISA titers of RBD-specific antibodies, adjuvants significantly increased titers (Figure 3b). AddaVax was identified as the most effective adjuvant. There was a gradual, significant increase in ELISA antibody titers after each of the first and second boosts. Specifically, ELISA titers after the second boost with RBD + adjuvant were as follows: 1.3 × 10^5^, RBD + AddaVax; 7.3 × 10^4^, RBD + alhydogel; and 4.1 × 10^4^, RBD + alum; while the mean titer of sera of the RBD group was approximately 1.3 × 10^4^ (Figure 3c). These results suggest that the RBD alone or with adjuvant-formulated RBDs elicited a strong and potent humoral response.

The Th1 and Th2 T helper (Th) lymphocyte subsets are associated with the production of IgG2a and IgG1, respectively [58,59]. The ratio of IgG1 to IgG2a indicates the balance between cell-mediated and humoral immunity [60]. To determine the type of immune response induced by plant-based RBD, we measured IgG1 and IgG2a levels in sera collected after the third immunization. The sera from the mice immunized with RBD alone or with RBD formulated with adjuvants had high anti-IgG1 titers against RBD, and in contrast, IgG2a titers were essentially undetectable (Figure 3d,e). These results indicate an induction of Th2-type humoral immune response by plant-based RBD in mice.

**Figure 4:**
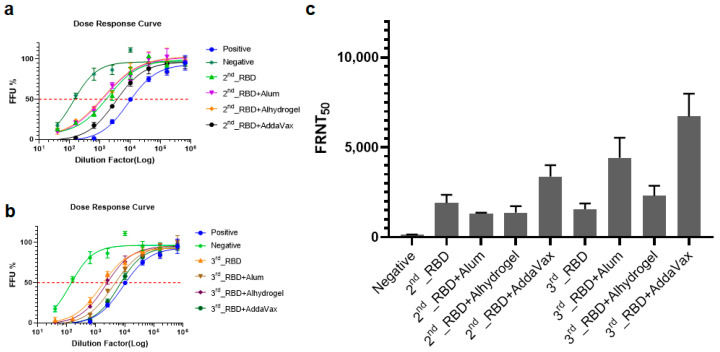
Virus-specific neutralization assay: (**a**,**b**) Dose–response curves of neutralizing activity of the second (**a**) and third (**b**) collections of sera. (**c**) Comparison of 50% focus reduction neutralization tests (FRNT_50_) values for each group.

To determine the virus-neutralizing activities of the mouse antisera, we performed FRNT assays of the four groups of sera collected after the second and third boosts using Vero E6 cells infected with live SARS-CoV-2 (Figure 4a,b and Figure S3). The mean FRNT_50_ value of sera collected from mice immunized with plant-based RBD after the second boost was 1843, which was significantly higher compared with those of the other groups (Figure 4c). Furthermore, the titers of neutralizing antibodies increased when mice were immunized with the other three adjuvants plus the RBD. After the third immunization, there were no significant differences in the titers of neutralizing antibodies of the plant-based RBD-only group. Moreover, the neutralizing titers of all groups immunized with RBD plus adjuvants significantly increased (Figure 4c and Figure S3), and among the three adjuvants, RBD formulated with AddaVax achieved the highest neutralizing mean antibody titer (FRNT_50_ = 6412). Together, these results demonstrate that plant-based RBD induced RBD-specific antisera in mice that protected Vero E6 cells from SARS-CoV-2 infection, highlighting the potential of plant-expressed RBD as an effective vaccine.

## 4. Discussion

The prevalence of the COVID-19 pandemic for a second year with an increasing worldwide infection rate has inflicted unimaginable social and economic losses. The highly effective SARS-CoV-2 vaccines introduced in late 2020 require storage at low temperatures, making it very difficult and expensive to administer mass vaccinations to the populations of resource-limited countries [61]. In contrast, plant-based transient recombinant protein production offers a significantly less expensive, safe, robust, and rapid production platform to produce future COVID-19 vaccines, particularly in emergencies, such as to combat infections with new SARS-CoV-2 variants. The SARS-CoV-2 RBD is an attractive target for subunit vaccine development, because it interacts with the host receptor ACE2 during virus entry into host cells. SARS-CoV-2 spike protein containing RBD is the target antigen of the currently available vaccines Pfizer/BioNtech Comirnaty, AstraZeneca/AZD1222 vaccines, Janssen/Ad26.COV 2.S, Moderna mRNA 1273 vaccine, and Sputnik V vaccine [62]. Here we expressed the RBD of the SARS-CoV-2 spike protein in a tobacco plant to develop a subunit vaccine. We show that the RBD was expressed in *N. benthamiana* within 4 days after agroinfiltration. The transient expression system used to produce RBD antigen is viable for commercial production, particularly in the emergency such as the current SARS-CoV2 pandemic, compared to a stable expression system that requires several months to years to generate the whole plant expressing the target antigen.

Increasing gene transcription represents an effective strategy to express high yields of proteins in plants. For example, the LS in the 5′-UTR sequence of the coronavirus genome plays a crucial role in gene expression during replication, and silencing the LS using an siRNA strongly inhibits SARS-CoV replication [63,64]. Thus, we employed the LS to enhance the expression level of RBD in *N. benthamiana*, and added LS in the MSP to improve RBD yields (Figure 1a). However, RBD yields were not enhanced by LS sequences (Figure 1c). The expression level of soluble RBD in crude extract was 92 mg/kg fresh leaf weight vs. 8 mg/kg fresh weight previously reported [65]. The differences in RBD expression level in our and previous studies may be attributed to the different vectors used and the different methods used to quantify the expressed protein.

Scalability is a limitation of translating a vaccine from the laboratory to commercial manufacturing, and the yield, production time, and cost determine the scalability of any vaccine manufacture. In contrast to the several years required for production of a vaccine in a stable transgenic plant, due to the requirement of regeneration of the whole plant from transgene integrated plant cells or tissues [66], our transient expression system produced research-grade RBD antigen in less than a month after the RBD nucleotide sequence became available, which is consistent with reports by others [37,50]. The feasibility of vaccine production in bacteria and mammalian cells is limited by the huge investment for maintaining sophisticated culture system [67]. Our transient system requires only inexpensive nutrient solutions and the space to grow the host plant for 4–5 weeks, which significantly reduces the expense of industrial-scale production compared with that for purchasing large volumes of specialized media required for the microbial and mammalian production system. Thus, the fast production and low cost for amplifying the vaccine production by the plant-based transient expression system in tobacco plant ensure the scalability.

The increased molecular mass of plant-based RBD compared with that predicted from its amino sequence (35 kDa vs. 27 kDa, respectively) can likely be attributed to glycosylation at two N-glycosylation sites (Asp) in the RBD sequence when analyzed with N-glycosylation prediction server NetNGlyc 1.0 http://www.cbs.dtu.dk/services/NetNGlyc/ (accessed on 12 June 2021) and the presence of 10 O-linked glycosylation sites [51,68]. The RBD protein bands in between 25 kDa and 35 kDa were consistently detected (Figure 2b), leading us to conclude that N- and O-glycation heterogeneity accounted for these findings [69]. Furthermore, the 64 kDa band detected in the Western blots of purified RBD (Figure 2b,c) likely represents a dimer, because the RBD contains nine cysteine residues, and such a dimer is known [56].

In previous studies, RBD was expressed in plants and evaluated only in vitro activities in binding to ACE2 and antibodies from COVID-19 convalescent plasma to develop the RBD antigen for use as a diagnostic marker [65,70]. Here, we report in vivo functional study of plant-expressed RBD by mice immunization and demonstrate the neutralization potency of the induced RBD specific antibodies in neutralizing the SARS-CoV-2 virus in vitro. The results from in vivo testing of novel vaccine candidate in the model animal makes a critical decision in translating the basic vaccine research into clinical application [71]. Mouse is a well-established animal model for the study of human disease and the efficacy of candidate drugs and vaccines [72]. The immune systems of mice have been well characterized, and a large number of immunological reagents are available for the analysis of the immune response [71]. Hence, in our study, we used mice strain BALB/cAnHsd, an excellent responder to immunization [73]. Here, we showed that plant-based RBD can likely serve as an effective vaccine, because it induced high-titer humoral immune responses in mice and that these anti-RBD sera protected Vero E6 cells from SARS-CoV-2 infection.

The S protein of SARS-CoV-2 is highly glycosylated, with 22 N-linked glycosylation sites [74,75]. N-linked glycans are critical for proper folding of the peptide chain and for inducing neutralizing antibodies [13,75]. N-glycosylation sites and glycosylation patterns affect the immunogenicity of antigens, and the glycoengineering of the host plant-based RBD confers increased immunogenicity in vivo [76,77]. Moreover, the glycosylation of a viral protein may mask epitopes that induce neutralizing or protective antibodies. For example, removal of N-glycosylation sites increases the accessibility of the immune response to hidden neutralizing or T-cell epitopes in the gp120 envelope glycoproteins of human and simian immunodeficiency viruses [78,79,80,81]. Structural studies have shown that the S protein is uniformly glycosylated with smaller glycans, which provides a larger surface accessible to antibodies compared with that of the S protein glycosylated with complexed glycans [82]. Thus, we used the glycoengineered -FX tobacco plant as a host to produce a humanized RBD with the expectation of improved antibody binding capacity and neutralizing efficacy through removal of plant-specific glycans. Furthermore, we found that the plant-based RBD antigen induced virus-neutralizing antibodies in mice that efficiently protected Vero E6 cells from virus infection. Based on our experience with the -FX tobacco plant, in future we will compare the humoral and cellular responses of plant-based RBD produced in wild-type and -FX tobacco plants.

We show here the ability of plant-based RBD to induce RBD-specific neutralizing antibodies in mice. Typically, robust production of a subunit vaccine requires adjuvant. Alum and alhydrogel adjuvants are generally used to induce a Th2-type immune response. MF59-like AddaVax, an oil-in-water emulsion-based adjuvant, stimulates both Th1/Th2 response [83,84]. In our study, plant-based RBD without adjuvant was sufficient to induce the RBD-specific antibodies in mice after a second booster dose (Figure 3a). Consistent with the immune response to recombinant RBD antigen reported by others, plant-based RBD induces a humoral immune response [68,85,86,87]. Here we show that immunization of mice with plant-based RBD induced high-titer RBD-specific neutralizing antibodies that protected mice from infection with SARS-CoV-2.

## 5. Conclusions

Recombinant RBD antigen produced in glycoengineered *N. benthamiana* induced antibodies and elicited a humoral immune response in mice. Moreover, these antibodies potently neutralized live SARS-CoV-2 in-vitro. These findings provide compelling evidence that this plant-based protein production system will effectively and inexpensively produce subunit vaccines against SARS-CoV-2.

## Figures and Tables

**Table 1:** Vaccination scheme.

No.	Group	Mouse No.	Antigen	Adjuvant	Total Volume	Immunization Route
1	PBS	5	-	-		
2	RBD only	5	10 μg of RBD	-	50 µL	I.M.
3	RBD + Alum	5	10 μg of RBD	Alum		
4	RBD + Alhydrogel	5	10 μg of RBD	Alhydrogel		
5	RBD + AddaVax	5	10 μg of RBD	AddaVax		
6	Alum only	5	-	Alum		
7	Alhydrogel only	5	-	Alhydrogel		
8	AddaVax only	5	-	AddaVax		

## Data Availability

Data are contained within the article.

## Supplementary Materials

The following are available online at https://www.mdpi.com/article/10.3390/vaccines9090978/s1, Figure S1: Detection of plant-based SARS-CoV-2 RBD protein expressed in glycoengineered *Nicotiana benthamiana* using different constructs (T10, T11, T12, T13). The protein was extracted from the tobacco leaves harvested 4 days post infiltration (4 DPI). The immunoblot membrane was probed with a rabbit polyclonal anti-SARS-CoV-2 (COVID-19) Spike RBD antibody. The number below the protein band represents the densitometric intensity ratio of the protein in each lane. Abbreviations: M, protein size marker; R, 100ng of SARS-CoV-2 RBD protein from Expi293F cells (positive control); N, non-infiltrated (negative control), Figure S2: Detection of purified plant-based SARS-CoV-2 RBD protein in immunoblot proved with probed with rabbit polyclonal anti-SARS-CoV-2 (COVID-19) Spike RBD antibody (a) and 6xHis HRP antibody. The number above or below the protein band represents the densitometric intensity ratio of the protein in each lane. M, protein size marker; Lane 1, non-infiltrated total soluble protein (TSP); Lane 2, RBD-infiltrated TSP; Lane 3, recombinant RBD purified from tobacco plant leaves, Figure S3: Representative panel of FRNT assay to demonstrate the potency of plant-based RBD immunized mouse sera to neutralize SARS-CoV-2. Sera samples were obtained from the mice immunized three times with plant-based RBD alone or RBD with adjuvants. The number of infected cells was counted and calculated FRNT^50^ as described in materials and methods.
