# Supplementary material for: Plant-Expressed Receptor Binding Domain of the SARS-CoV-2 Spike Protein Elicits Humoral Immunity in Mice
Source: Vaccines (Basel). 2021 Sep 1;9(9):978. doi: 10.3390/vaccines9090978 (PMC8472882; doi:10.3390/vaccines9090978)
Supplement: Supplementary file 1 [file vaccines-09-00978-s001.zip › vaccines-1330131-supplementary.pptx]

## Slide 1
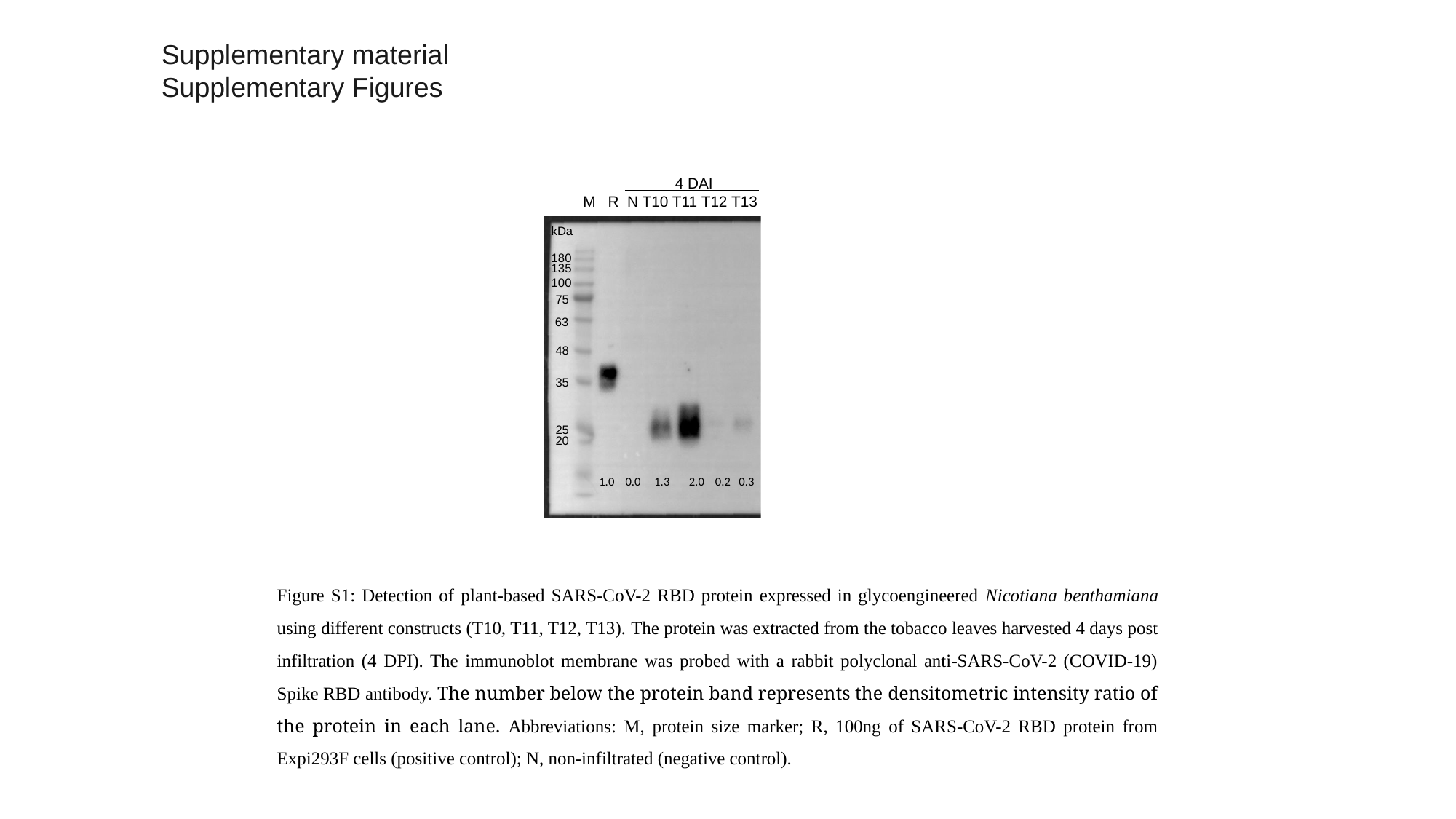

Supplementary material
Supplementary Figures
 4 DAI
 M R N T10 T11 T12 T13
kDa
180
135
100
75
63
48
35
25
20
1.0 0.0 1.3 2.0 0.2 0.3
Figure S1: Detection of plant-based SARS-CoV-2 RBD protein expressed in glycoengineered Nicotiana benthamiana using different constructs (T10, T11, T12, T13). The protein was extracted from the tobacco leaves harvested 4 days post infiltration (4 DPI). The immunoblot membrane was probed with a rabbit polyclonal anti-SARS-CoV-2 (COVID-19) Spike RBD antibody. The number below the protein band represents the densitometric intensity ratio of the protein in each lane. Abbreviations: M, protein size marker; R, 100ng of SARS-CoV-2 RBD protein from Expi293F cells (positive control); N, non-infiltrated (negative control).

## Slide 2
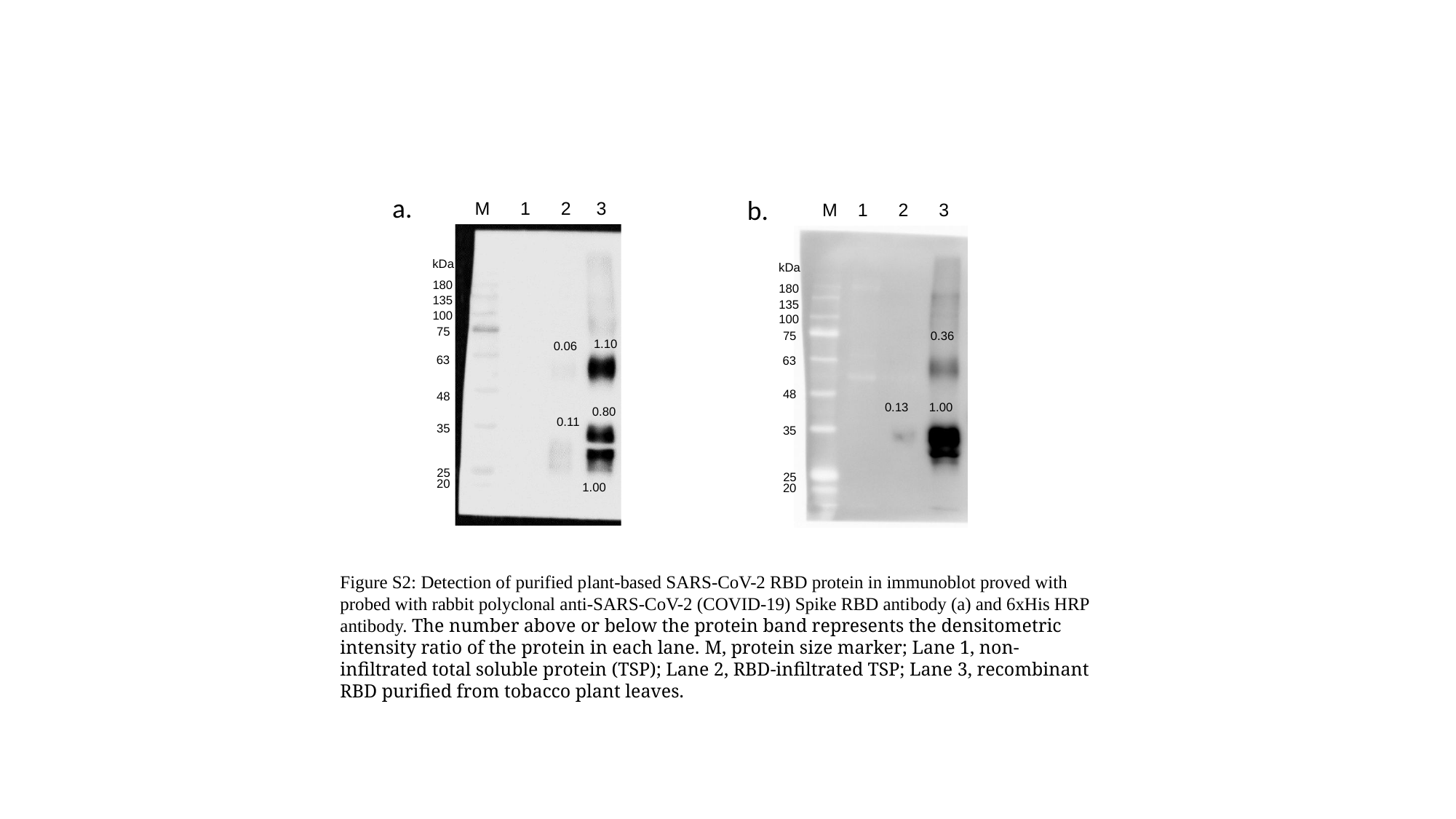

a.
b.
 M 1 2 3
 M 1 2 3
kDa
kDa
180
180
135
135
100
100
75
75
0.36
1.10
0.06
63
63
48
48
0.13
1.00
0.80
0.11
35
35
25
25
20
1.00
20
Figure S2: Detection of purified plant-based SARS-CoV-2 RBD protein in immunoblot proved with probed with rabbit polyclonal anti-SARS-CoV-2 (COVID-19) Spike RBD antibody (a) and 6xHis HRP antibody. The number above or below the protein band represents the densitometric intensity ratio of the protein in each lane. M, protein size marker; Lane 1, non-infiltrated total soluble protein (TSP); Lane 2, RBD-infiltrated TSP; Lane 3, recombinant RBD purified from tobacco plant leaves.

## Slide 3
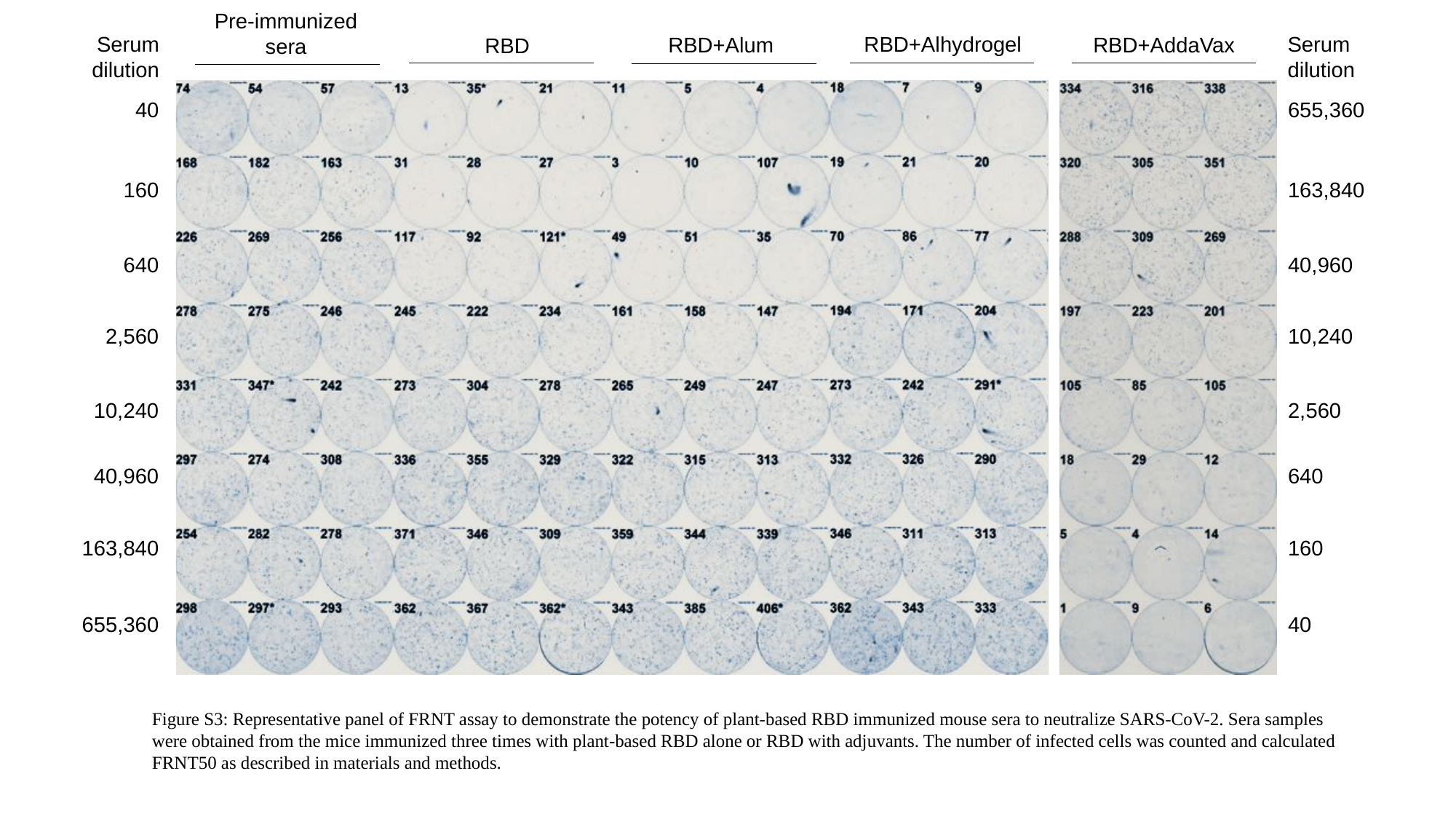

Pre-immunized
sera
Serum dilution
RBD+Alhydrogel
Serum dilution
RBD+AddaVax
RBD+Alum
RBD
40
655,360
160
163,840
640
40,960
2,560
10,240
10,240
2,560
40,960
640
163,840
160
655,360
40
Figure S3: Representative panel of FRNT assay to demonstrate the potency of plant-based RBD immunized mouse sera to neutralize SARS-CoV-2. Sera samples were obtained from the mice immunized three times with plant-based RBD alone or RBD with adjuvants. The number of infected cells was counted and calculated FRNT50 as described in materials and methods.
